# Development and Validation of a Social Capital Questionnaire for Adolescent Students (SCQ-AS)

**DOI:** 10.1371/journal.pone.0103785

**Published:** 2014-08-05

**Authors:** Paula Cristina Pelli Paiva, Haroldo Neves de Paiva, Paulo Messias de Oliveira Filho, Joel Alves Lamounier, Efigênia Ferreira e Ferreira, Raquel Conceição Ferreira, Ichiro Kawachi, Patrícia Maria Zarzar

**Affiliations:** 1 Department of Child and Adolescent Health, Universidade Federal de Minas Gerais, Belo Horizonte, Brazil; 2 Department Dentistry, Universidade Federal dos Vales do Jequitinhonha e Mucuri, Diamantina, Brazil; 3 Department of Basic Sciences, Universidade Federal dos Vales do Jequitinhonha e Mucuri, Diamantina, Brazil; 4 Department of Oral Public Health, School of Dentistry, Universidade Federal de Minas Gerais, Belo Horizonte, Brazil; 5 Department of Social and Behavioral Sciences, Harvard School of Public Heath and Medical School, Harvard, Boston, Massachusetts, United States of America; 6 Pediatric Dentistry and Orthodontics, School of Dentistry, Universidade Federal de Minas Gerais, Belo Horizonte, Brazil; Iranian Institute for Health Sciences Research, ACECR, Islamic Republic Of Iran

## Abstract

**Objectives:**

Social capital has been studied due to its contextual influence on health. However, no specific assessment tool has been developed and validated for the measurement of social capital among 12-year-old adolescent students. The aim of the present study was to develop and validate a quick, simple assessment tool to measure social capital among adolescent students.

**Methods:**

A questionnaire was developed based on a review of relevant literature. For such, searches were made of the Scientific Electronic Library Online, Latin American and Caribbean Health Sciences, The Cochrane Library, ISI Web of Knowledge, International Database for Medical Literature and PubMed Central bibliographical databases from September 2011 to January 2014 for papers addressing assessment tools for the evaluation of social capital. Focus groups were also formed by adolescent students as well as health, educational and social professionals. The final assessment tool was administered to a convenience sample from two public schools (79 students) and one private school (22 students), comprising a final sample of 101 students. Reliability and internal consistency were evaluated using the Kappa coefficient and Cronbach's alpha coefficient, respectively. Content validity was determined by expert consensus as well as exploratory and confirmatory factor analysis.

**Results:**

The final version of the questionnaire was made up of 12 items. The total scale demonstrated very good internal consistency (Cronbach's alpha: 0.71). Reproducibility was also very good, as the Kappa coefficient was higher than 0.72 for the majority of items (range: 0.63 to 0.97). Factor analysis grouped the 12 items into four subscales: School Social Cohesion, School Friendships, Neighborhood Social Cohesion and Trust (school and neighborhood).

**Conclusions:**

The present findings indicate the validity and reliability of the Social Capital Questionnaire for Adolescent Students.

## Introduction

Social capital is defined as the resources generated by the participation of individuals in social networks as well as the norms of trust and reciprocity that emerge from these interactions [1]. The concept has both a cognitive component (e.g. perceptions of the trustworthiness of the group) as well as a structural component (the density or frequency of social participation) [2]. Growing evidence points to social capital as a determinant of health [3].

Social capital research has emerged as a focus of contemporary behavioral epidemiology due to the need for more effective measures aimed at increasing protective health behaviors and decreasing risk behaviors. Therefore, social capital has become particularly important, as social contexts can influence health-related behaviors [4]. Considered crucial to the functioning of life in the community through a variety of domains, social capital encompasses the prevention of delinquency and criminal behavior among adolescents and young people, the promotion of youth development, respect for schools, education and democracy and the advancement of economic development [1], [5]. Adolescents are at a stage of development in which they become decreasingly dependent on family and increasingly involved in the establishment of peer networks. Social capital can influence health through the exercise of collective actions and informal social control [6]. The challenge in social capital research is to identify and isolate the specific instances in which it can be a driving force for health promotion versus a detrimental factor in the health status of adolescents [6], [7].

The bulk of empirical studies to date have been conducted using adult samples and less is known regarding the links between social capital, wellbeing and health outcomes among adolescents. Even when adolescents are the object of investigation, studies describe findings based on questionnaires originally designed for adults, often completely overlooking the perceptions of adolescents as active social agents [8]. Indeed, the literature on assessment tools developed to evaluate social capital among younger adolescents is scarce.

In the United States of America (USA), all 31 scales designed to measure social capital have been developed for adults [8]. Recent papers have been published on the development of a short version of the Personal Social Capital Scale as well as the Social Capital Investment Inventory. However, both assessment tools have been developed and validated for the adult population [10], [11]. Moreover, a search of the databases in 2011 also turned up no assessment tools for measuring social capital designed for children and adolescents that simultaneously addressed social networks, social cohesion, sense of belonging, trust, autonomy and control. The few studies on social capital in this population employ only one or two questions about one's network of friends or neighborhood cohesion, e.g., *Our school is a nice place to be*, *I feel I belong in this school*, *I feel safe in this school*, *You can trust people around here*, *I could ask for help or a favor from my neighbors*, *Most people in this neighborhood are willing to help you in case of need* and *In this neighborhood, you have to be careful or someone is likely to take advantage of you* [6], [8], [12].

As a result, the methodology for measuring social capital is still debated in the epidemiological literature [13]. According to Bowling [14], there is currently no scale considered the gold standard for evaluating the major domains of social capital with acceptable levels of reliability and validity.

According Streiner and Norman [15], the development of a valid, reliable assessment tool is not a trivial task and the use of previously designed and tested instruments is recommended. Considering the importance of this concept in the investigation of risk behavior and health determinants among adolescents, a questionnaire that encompasses the different domains of social capital is needed for this population.

The aim of the present study was to develop a questionnaire for measuring social capital that is easily understood and applicable to adolescent students. We further sought to evaluate the content validity and psychometric reliability of our novel instrument.

The use of a social capital assessment tool specifically designed for adolescents can assist in the investigation and understanding of behaviors related to health and wellbeing. Such an assessment tool should measure contexts that involve interrelationships, such as experiences at school and in the local community, which can exert an influence on the behavior and decisions of adolescents, thereby reflecting health determinants.

## Methods

The present cross-sectional study was carried out in southeastern Brazil in a municipality with 46,372 inhabitants, an 83.4% literacy rate, a human development index (HDI) of 0.748 and an income HDI of 0.752. A total of 7474 schoolchildren are enrolled in elementary schools in urban and rural areas of the municipality (477 in private schools and 6997 in public schools) [16]. The study population included a convenience sample made up of 101 students aged 12 years enrolled in the public and private school systems. The development of the Social Capital Questionnaire for Adolescent Students was carried out in two phases: 1) the development of the questionnaire and 2) the evaluation of its psychometric properties. The data were collected in September and October 2012.

### Development of the questionnaire

#### Conceptual basis, design and adaptation of items

Searches were carried out between September and November 2011 in the Indexpsi Pepsic, SciELO, Lilacs, Index Psi TCCs, Cochrane Library and Pubmed databases for publications in the past 20 years using the key words *child, adolescent, schoolchildren, social capital, social network, social cohesion, validation and development of instruments*. Two researchers (one with experience in the validation of assessment tools for adolescents and the other with extensive knowledge on studies addressing social capital) then held meetings to draft the questionnaire (PMZ, EFF), establishing the content of the items and constructs of the questionnaire. After defining the items, the questionnaire was submitted to the conceptual evaluation of an expert in social capital (IK). The resulting version was evaluated by three researchers for the semantic equivalence, conceptual scope, clarity, relevance and conciseness of each item: one researcher with experience in child/adolescent health and the development/validation of instruments; one researcher with experience in public health and child/adolescent health; and one researcher with experience in public health, adolescent health and social capital (EFF, DG, MLRJ) [17], [18]. The opinions of the three researchers were compiled and no need for further changes was deemed necessary. The questionnaire was then sent to a linguistics professor for the verification of grammar.

#### Face validity - Adolescent focus group

The questionnaire was discussed in a focus group composed of a convenience sample of 12 adolescents (six from public schools and six from private schools) to identify their understanding of the items on the questionnaire. The schools and participants were chosen by convenience. The following were the inclusion criteria for participation in the adolescent focus group: regular enrollment in school; 12 years of age; authorization from parents through a signed statement of informed consent; and agreement to participate.

Meetings with the focus group occurred on two occasions. The first meeting was held to sensitize the students and distribute the questionnaires. The adolescents were instructed to take the questionnaire home and answer it without the assistance of anyone and write down any questions that arose during the process to be discussed at the following meeting. The second meeting was held to discuss all items on the questionnaire using a brainstorming technique for the analysis of the comprehension of each item, in which the participants expressed all their ideas regarding the item, including their questions and suggestions for changes. The aim of this process was to analyze the relevance of the items, determine whether additional items should be included and evaluate the comprehensibility and applicability of the questionnaire to children/adolescents.

The discussion of the focus group ended after about 60 minutes, when all items had been discussed exhaustively. The researcher conducted the meeting, which was recorded and later transcribed. The observations and suggestions of the participants were recorded. The recordings of both meetings were transcribed and analyzed. The observations were grouped and the procedure was repeated for the suggestions. Each category was classified as *difficult to understand* or *conceptual problem*. After this analysis, the questionnaire was revised with the addition of the suggestions proposed by the adolescents and sent to external two researchers [one with experience in the validation of assessment tools among adolescents and the other with extensive knowledge on studies addressing social capital (PMZ and EFF)] for consideration.

#### Face validity - Adult focus group

After input from the adolescents and the consideration of the reviewers, the questionnaire was submitted to an adult focus group, which was made up of two parents, three educators, a sociologist, two psychologists, a philosopher and a social worker. The adult focus group met to evaluate the questionnaire with regard to congruence, clarity and conceptual scope. The suggestions were recorded and sent to the reviewers (PMZ and EFF) for consideration.

### Content validity

The qualitative analysis of the questionnaire was performed by two researchers experienced with validation studies (EFF and PMZ) for the analysis and revision of the questionnaire, arriving at the final version through consensus. After the application of minor changes suggested by the focus groups, the questionnaire was considered appropriate for use on the age group indicated.

### Evaluation of psychometric properties

#### Internal consistency and validation

The questionnaire was administered to a convenience sample made up of 101 12-year-old students [82.2% (n = 83) enrolled in public schools and 17.8% (n = 18) enrolled in private schools]. The questionnaire was self-administered in the classroom setting. A researcher read each item aloud to avoid bias stemming from differences in reading proficiencies among the participants. The aim of the self-administration of the questionnaire was to evaluate the ease of administration, the format and the time required to fill out the questionnaire. Reliability was tested through a second administration of the questionnaire to 50% of the sample after a two-week interval.

### Reliability

The reliability of the questionnaire was measured based on its reproducibility (test-retest stability) and internal consistency. Exploratory factor analysis was performed to evaluate the dimensional structure of the questionnaire.

Reliability (test-retest reproducibility) was evaluated through the calculation of weighted Kappa coefficients. The analysis of internal consistency and the behavior of each item determined the definition of the items to be maintained, with the removal of those with an item-to-total correlation of less than 0.20. The contribution of each item to the increase in Cronbach's alpha coefficient of the questionnaire was also determined.

For the evaluation of construct validity, the 12 items were submitted to exploratory factor analysis to determine the pattern of joint variation of the items and the variance explained by each factor. Conceptually defined latent dimensions formed by the items were identified. The determination of the adequacy of the factor analysis was performed through the analysis of the anti-image correlation matrix, Bartlett's test (to test the hypothesis of sufficient correlation among the variables) and the Kaiser Meyer-Olkin (KMO) measure. The KMO statistic ranges from 0 to 1, with values closer to 1 denoting greater adequacy of the factor analysis. Communalities represent the variance each item shares with other variables or items on a scale. Researchers generally consider an absolute load value >0.3 to be important, depending on the size of the sample. For a sample of 101 individuals, factor loadings >0.50 are considered significant [19]. Factors were extracted considering eigenvalues >1 and orthogonal rotation was performed using the Varimax method. If similar items exhibited loadings in different factors, they were allocated to the most appropriate factor based on the nature of the items.

CFA (confirmatory factor analysis) using structural equation models the method asymptotic distribuition free was carried for the whole sample to test the four factor structure identified in exploratory analysis (analysis principal components). The goodness of fit for the compenting models was evalued through fit indices: Root mean square error of approximation (RMSEA); comparative fit index (CFI); chi-square test.

Data analysis was performed using the SPSS version 17.0 (SPSS Inc., Chicago, IL, USA). The statistical software Stata version 12 (2011; Stata, College Station, Tex) was used to carry out the CFA.

### Ethical considerations

This study was approved by the Human Research Ethics Committee of the Federal University of Minas Gerais (Brazil) (COEP-317/11). All parents/guardians signed a statement of informed consent authorizing the participation of their children. All adolescents also signed a statement of informed consent.

The table 1 summarizes of method employed for the development and validation of the questionnaire for measuring social capital among adolescent students.

**Table 1:** Summary of method employed for the development and validation of the questionnaire for measuring social capital among adolescent students.

Purpose	Steps	Participants
**Drafting of questionnaire**	Determine availability of adequate assessment tool that corresponds to study aims; Identify forms and possible sources of information in *PUBMED, Indexpsi Pepsic, SciELO, Lilacs, Index Psi TCCs, Cochrane Library* databases for definition of items on questionnaire	Authors Researchers
**Conceptual basis, drafting and adaptation of items**	Evaluate questionnaire with focus on conceptual scope, relevance, clarity and conciseness	Researchers and experts in field (consensus analysis)
**Face validity**	Investigate comprehension, congruence and applicability of questionnaire to children/adolescents	Adolescent focus group
		Researchers
	Remove or rewrite items that do not correspond to the objective or those that were not well understood	Experts in field
	Investigate comprehension, semantics, clarity and conceptual scope	Adult focus group
**Content validity**	Evaluate final version of questionnaire following focus group interviews (consensus analysis)	Experts in field
**Evaluation of psychometric properties**	Test questionnaire	Population sample
	Internal consistency and validation	
	Reliability	
**Validity analysis methods**	Test-retest stability	
	Exploratory factor analysis	
	Kappa coefficients	
	Confirmatory factor analysis	

## Results

The draft of the questionnaire after linguistic revision was made up of 16 items. Each item had response options in the form of a Likert scale. Agreement with negative statements and disagreement with positive statements received a score of 1, whereas agreement with positive statements and disagreement with negative statements received a score of 3. This version of the questionnaire demonstrated adequate face validity, as it was well understood by the adolescents. Only minor semantic changes were deemed necessary when the items were discussed one by one. The adult focus group suggested minor semantic changes, such as that applied to Item 2, to which the expression *as if it were mine* was added. The questionnaire demonstrated adequate content validity, as attested by the experts, who concluded that it incorporated all the domains of interest.

A total of 46.5% of the participants (n = 47) were male. A total of 17.8% (n = 18) attended private schools and 82.3% (n = 83) attended public schools. Mean time required to answer the 16-item questionnaire was 21.0±4.09 minutes. The 16 items demonstrated good reproducibility, with weighted Kappa coefficients ranging from 0.635 to 0.971 (Table 2).

**Table 2:** Weighted Kappa coefficients of items on Social Capital Questionnaire for Adolescent Students divided into three subscales (sample of 101 12-year-olds), southeastern Brazil, 2013.

Items	*K*
**Social network/cohesion/sense of belonging**	
(1) The students at my school stay together.	0.887
(2) I feel like I belong at this school, as if it were mine.	0.820
(3) I feel safe at this school.	0.939
(4) The students at my school have fun together.	0.873
(5) When the students at my school are having fun, some are left out.	0.750
(6) Bullying occurs at my school.	0.724
(7) The teachers at my school are sympathetic and give us support.	0.155
(8) My parents get along with my teachers.	0.743
**Trust**	
(9) I trust my neighbors.	0.942
(10) I can count on my neighbors for help.	0.784
(11) My neighbors would try to take advantage of me.	0.690
(12) My classmates would try to take advantage of me.	0.818
(13) I trust my friends at school.	0.635
(14) I can ask my friends at school for help.	0.850
**Autonomy and Control**	
(15) My mother controls everything I do.	0.969
(16) My father controls everything I do.	0.971

The 12-item questionnaire demonstrated item-total correlations ranging from 0.225 to 0.427 and Cronbach's alpha coefficient for the total scale was 0.707 (Table 3). The four items excluded referred to bullying and parental control. As the subscale *Autonomy and Control* was composed of only two items, which did not demonstrate satisfactory internal consistency, the decision was made to exclude it. The 12-item questionnaire resulted in a score ranging from 12 to 36 (sum of each item score), with higher scores denoting a higher level of social capital.

**Table 3:** Internal consistency of items on Social Capital Questionnaire for Adolescent Students divided into four subscales (sample of 101 12-year-olds), southeastern Brazil, 2013.

	16 items		12 items	
	Corrected Item-Total Correlation	Cronbach's Alpha if Deleted	Corrected Item-Total Correlation	Cronbach's Alpha if Deleted
The students at my school stay together?	0.298	0.635	0.322	0.682
I feel like I belong at this school, as if it were mine.	0.373	0.622	0.344	0.679
I feel safe at this school.	0.390	0.620	0.376	0.674
The students at my school have fun together.	0.249	0.642	0.294	0.686
When the students at my school are having fun, some are left out.	0.096	0.657	-	-
Bulling occurs at my school.	0.075	0.657	-	-
The teachers at my school are sympathetic and give us support.	0.226	0.647	0.225	0.694
My parents get along with my teachers.	0.418	0.625	0.429	0.672
I trust my neighbors.	0.354	0.626	0.376	0.673
I can count on my neighbors for help.	0.353	0.626	0.378	0.673
My neighbors would try to take advantage of me.	0.302	0.634	0.326	0.682
My classmates would try to take advantage of me.	0.197	0.654	0.231	0.700
I trust my friends at school.	0.369	0.624	0.391	0.672
I can ask my friends at school for help.	0.370	0.624	0.363	0.676
My mother controls everything I do.	0.007	0.671	-	
My father controls everything I do.	0.078	0.667	-	

Exploratory factor analysis of the 12-item questionnaire identified four subscales: school social cohesion, school friendships, neighborhood cohesion and trust (school and neighborhood). These four subscales explained 61.68% of the variance in the data. The first factor alone explained 24.14% of the overall variability in the data. The first two factors together explained 38.67% of the overall variability, and so on. Communalities ranged from 0.46 to 0.513 (Table 4). The KMO statistic was 0.63 and the result of Bartlett's test was <0.001.

**Table 4:** Factor loadings of items on Social Capital Questionnaire for Adolescent Students divided into four factors of alternative model; exploratory factor analysis through principal components with varimax rotation (sample of 101 12-year-olds), southeastern Brazil, 2013.

Item	Factors				Communality
	1	2	3	4	
**School cohesion (score: 4 to 12)**					
(1) The students at my school stay together;	**0.689**	0.064	0.089	−0.061	0.491
(2) I feel like I belong at this school, as if it were mine.	**0.684**	−0.019	0.248	−0.060	0.532
(3) I feel safe at this school.	**0.742**	0.082	0.007	0.122	0.573
(4) My parents get along with my teachers.	**0.621**	0.174	−0.034	0.381	0.563
**School friendships (score: 3 to 9)**					
(5) The students at my school have fun together.	0.429	**0.487**	−0.248	−0.194	0.460
(6) I trust my friends at school.	0.175	**0.819**	−0.025	0.129	0.719
(7) I can ask my friends at school for help.	−0.018	**0.890**	0.099	0.080	0.808
**Neighborhood social cohesion (score: 2 to 4)**					
(8) I trust my neighbors.	0.176	0.043	**0.850**	−0.024	0.756
(9) I can count on my neighbors for help.	0.091	0.007	**0.860**	0.135	0.765
**Trust: school/neighborhood (score: 3 to 9)**					
(10) The teachers at my school are sympathetic and give us support.	0.262	−0.093	−0.102	**0.744**	0.641
(11) My neighbors would try to take advantage of me.	−0.063	0.094	0.468	**0.590**	0.580
(12) My classmates would try to take advantage of me.	−0.209	0.357	0.204	**0.548**	0.513
**Eigenvalue**	2.89	1.74	1.60	1.15	
**% of variance explained**	24.14	38.67	52.08	**61.68**	

Loadings greater than 0.4 in bold type; Items in bold type: application of exploratory factor analysis.

**Figure 1:**
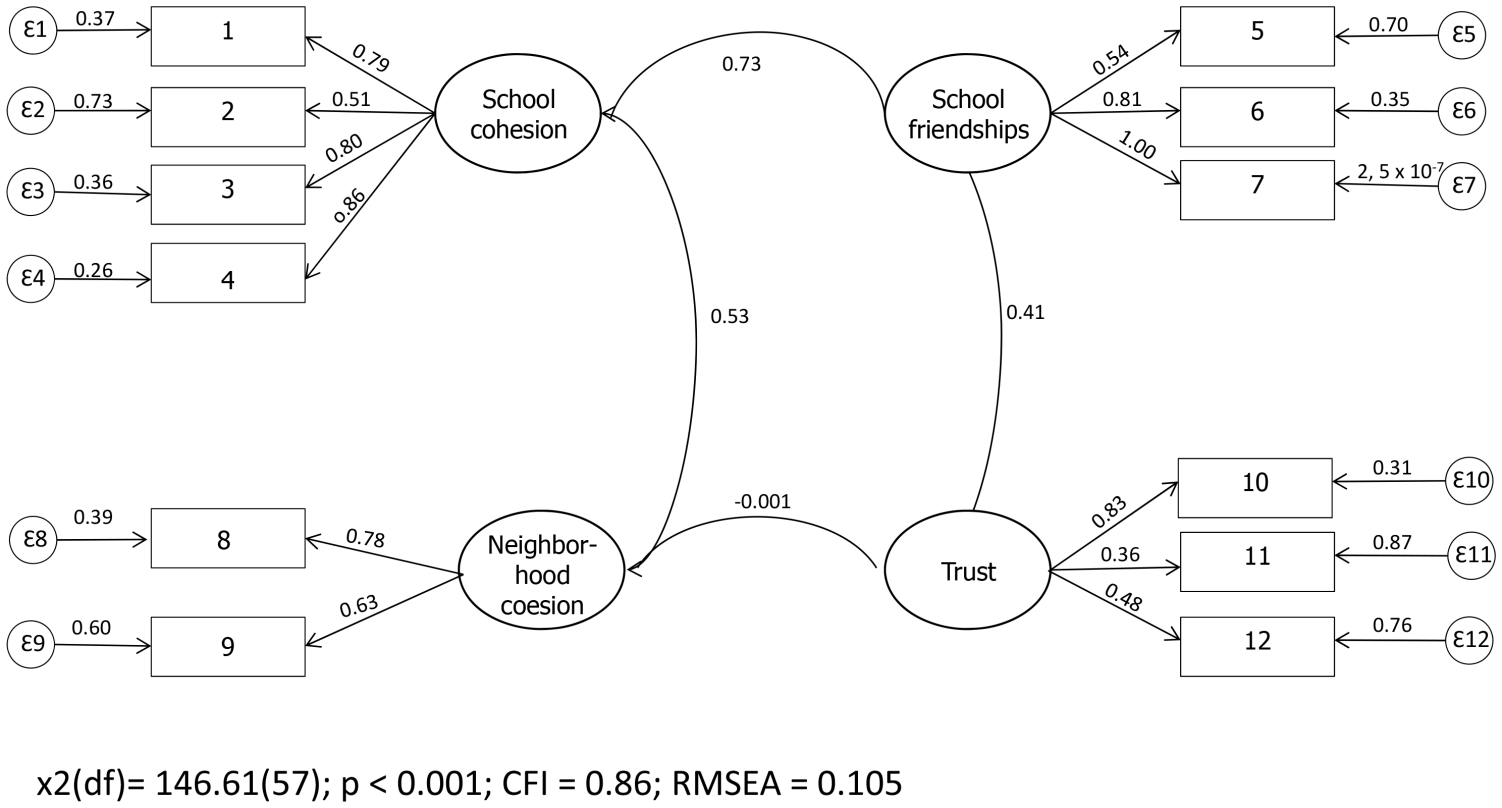
Measures of internal consistency of the construct by CFA for the alternative model; (sample of 101 12-year-olds), southeastern Brazil, 2013.

The four constructs were confirmed, with the items presenting CF from 0.36 to 1.0. The construct *Trust* presented the low value (Figure 1).

## Discussion

Most assessment tools used to measure social capital have been developed in English-speaking countries for the adult population and may therefore be susceptible to the influence of local culture or the local context. Consequently, some items may not accurately measure the domains of social capital in younger populations. Thus, an assessment tool designed to measure social capital specifically among adolescents is needed. Instruments that aim to measure social capital need to be validated prior to being administered to other populations, with cross-cultural adaptation while maintaining the context of the original assessment tool. Once these requirements have been met, the questionnaire can be administered to another population and furnish representative data on a given community [20].

The review of the literature identified 497 articles addressing social capital and its association with health conditions, such as morbidity and mortality rates, self-rated health status, wellbeing, heart disease, mental illness, obesity, stress, substance abuse and HIV [1], [5], [9], . The results of the literature review led to the selection of the subscales for the questionnaire to encompass social networks: social cohesion, sense of belonging and trust, and autonomy and control.

Adolescents spend most of their time involved in activities related to school, neighborhood and family settings. Thus, the questionnaire developed herein was based on relationships linked to these settings. Social capital in schools is an emerging topic of social capital research, since school may influence the health and wellbeing of adolescents [25]. The validation of a psychometric assessment tool is essential. The validity of a scale refers to the degree to which it measures the construct that it was developed to measure. The present questionnaire was submitted to both quantitative and qualitative analyses during its formulation and development [18], [26].

Trained professionals with experience in the development and validation of assessment tools analyzed the questionnaire with regard to the relevance of the items and pertinence of the construct. This procedure fulfilled the steps required for the theoretical analysis and refinement of the questionnaire [27].

The use of focus groups for the development of the theoretical reference was an important aspect of the present study, as the interviews were fundamental to the establishment of the face validity of the items.

The validation of the questionnaire was achieved through construct validation, the aim of which is to make a measure operational [28]. Exploratory factor analysis was the method of choice for this purpose [29]–[33]. This form of analysis is often employed when the primary objective is to measure a construct adequately. Exploratory factor analysis involves the grouping of variances. In the present study, this process detected consistency in four grouped factors that explained 61.68% of the overall variability in the data. These factors were interpreted based on that which the items had in common. The percentage of explanation of the overall variability was comparable to other validated instruments described in the literature [30], [34], [35].

The aim of the present study was to develop the Social Capital Questionnaire for Adolescent Students (SCQ-AS) and acquire evidence of its dimensional structure and reliability. Adequate internal consistency was found for each of the subscales as well as the overall scale. The analyses identified coherent, relevant dimensions. After the removal of four items from the initial questionnaire (*When the students at my school are having fun, some are left out*, *Bullying occurs at my school*, *My mother controls everything I do* and *My father controls everything I do*), the previously established subscales were confirmed. The reasons for the inadequate internal consistency of the aforementioned items may be due to difficulties in interpreting the statements stemming from the phase of cognitive development of the participants, who may have associated the item with the vigilance of conduct rather than a view of autonomy in relation to one's parents. The following are examples of commonly used forms in the literature to assess autonomy and control in adolescents: *How often does your mother or father try to control everything you do*?, *In our school, the students take part in making the rules*, and *My school provides me with the opportunity to be actively involved in decisions* [6], [8], [12].

Through the determination of the principal components, exploratory factor analysis demonstrated that the questionnaire had adequate psychometric properties in terms of construct validity. The factor loadings of the items related to each of the four factors were also satisfactory. The variance explained by the factor loadings was greater than 50% for the majority of items, which is similar to figures described in previous studies evaluating the reliability and construct validity of assessment tools employing the same type of analysis. The results were compatible with findings reported in other validation studies, in which communalities ranged from 0.40 to 0.90 [30], [31], [36].

Cronbach's alpha coefficient was used to evaluate the internal consistency of the questionnaire, which is the method employed in most validation studies found in the literature [31], [33], . Cronbach's alpha offers a statistical summary for the evaluation of agreement among all possible subsets of items, for which coefficients ≥0.70 are considered acceptable for comparisons among groups [40]. This method was chosen for the evaluation of the present questionnaire. The present findings are in agreement with data reported in previous studies, such as an investigation addressing an assessment tool developed to measure neighborhood quality of life in Taiwan, which had Cronbach's alpha coefficients ranging from 0.67 to 0.84 and factor analysis with varimax rotation explained 54.8% of the variance in the three subscales [34]. The findings are also in agreement with Cronbach's alpha coefficient in the analysis of the psychometric properties of the Brazilian version of the Early Childhood Oral Health Impact Scale, which was 0.74. The authors state that divergences in internal consistency may be found in distinct populations due to social and cultural differences. Accordingly, coefficients for the original English-language scale, the French version and the Chinese version were 0.91, 0.79 and 0.85, respectively [39].

Although there is no consensus regarding the effect of the number of categories of a scale on variability, which could compromise the reliability and validity of an assessment tool, it seems advisable to adopt a scale with a midpoint and clear, short statements that encompass the two extremes [41]. As a questionnaire designed for children and adolescents, the decision was made to use a three-point Likert scale with response options of *I agree*, *I do not agree or disagree* and *I disagree*. This procedure was based on the target age group and was chosen to avoid confusion during the filling out of the questionnaire.

The findings confirm indications in the literature that networks of friends and neighborhood cohesion reflect experiences one shares with one's peers and underscore the importance of the present questionnaire as an assessment tool for measuring social capital [8], [12], [42], [43].

Sapnas and Zeller [44] propose a sample size between 50 and 100 individuals to carry out factor analysis and evaluate the psychometric properties of questionnaires in the social sciences. In the present study, the sample was made up of 101 12-year-olds and normalization was performed using the KMO measure. Adherence of the present sample was 0.630, which is higher than the 0.60 recommended in the literature and demonstrates the goodness of fit of the data to factor analysis [45]. These findings are compatible with data reported in previous validation studies [46]–[48].

Although the potential influence of social capital on improvements in the health and wellbeing of adolescents is recognized, Aminzadeh et al. [8] point out that studies have not been able to employ an appropriate method for the analysis of its effects until only recently. Due to the cross-sectional design employed herein, causality cannot be inferred. However, the present findings suggest that the SCQ-AS demonstrates validity with regard to its dimensional structure, internal consistency and reliability, which encourages its use in further observational cohort studies. Indeed, this questionnaire has a number of advantages over other indices employed in the literature, since it was drafted to encompass the principal domains of social capital for a specific age group [49]. The fact that the sample was made up of adolescent students and the questionnaire had items that address one's neighborhood may also be considered advantages.

Further studies evaluating the use of the present questionnaire on children and adolescents are needed to demonstrate whether this assessment tool has the ability to discriminate groups with different ages and characteristics. Moreover, one should bear in mind that the present sample was restricted to one city in southeastern Brazil. Thus, the questionnaire should be administered to other populations. The validation of the SCQ-AS is hoped to stimulate the advance of studies in the field of social capital among children and adolescents and its positive effects on health.

We highlight our study as a pioneer in the development of an instrument to measure social capital in school adolescents. Up to date, we are developing a new study with a representative sample to confirm the results of present study.

## Conclusions

The findings of the present study offer evidence of the validity and reliability of the Social Capital Questionnaire for Adolescent Students (SCQ-AS) and demonstrate that this assessment tool is appropriate for epidemiological studies involving samples of adolescents in the investigation of the association between social capital and risk factors or health determinants.

### Ethical approval

This study obtained approval from the Research Ethics Committee of the Federal University of Minas Gerais (COEP-317/11).

## Supporting Information

File S1 **Social Capital Questionnaire for Adolescent Students (SCQ-AS).**
(DOC)
