# Supplementary material for: Development and Validation of a Social Capital Questionnaire for Adolescent Students (SCQ-AS)
Source: PLoS One. 2014 Aug 5;9(8):e103785. doi: 10.1371/journal.pone.0103785 (PMC4122396; doi:10.1371/journal.pone.0103785)
Supplement: File S1 — Social Capital Questionnaire for Adolescent Students (SCQ-AS). (DOC) [file pone.0103785.s001.doc]

**Social Capital Questionnaire for Adolescent Students (SCQ-AS)**

**HELLO,**

Thanks for agreeing to help us with our study!

This study is being done so that there will be more understanding about you and your health.

By answering the questions, you will help us learn more about young people’s experiences.

**PLEASE REMEMBER**

• Don’t write your name on the questionnaire

• This is not a test and there are no right or wrong answers

• Answer as honestly as you can. Don’t talk to anyone about the questions when you

are answering them. Your answers are private. No one you know will see them.

**School cohesion (score: 4 to 12)**

1. The students at my school stay together:

( ) Agree

( ) I do not know, have no opinion

( ) Disagree

2. I feel like I belong at this school, as if it were mine:

( ) Agree

( ) I do not know, have no opinion

( ) Disagree

3. I feel safe at this school:

( ) Agree

( ) I do not know, have no opinion

( ) Disagree

4. My parents get along with my teachers:

( ) Agree

( ) I do not know, have no opinion

( ) Disagree

**School friendships (score: 3 to 9)**

5. The students at my school have fun together:

( ) Agree

( ) I do not know, have no opinion

( ) Disagree

6. I trust my friends at school:

( ) Agree

( ) I do not know, have no opinion

( ) Disagree

7. I can ask my friends at school for help:

( ) Agree

( ) I do not know, have no opinion

( ) Disagree

**Neighborhood social cohesion (score: 2 to 4)**

8. I trust my neighbors:

( ) Agree

( ) I do not know, have no opinion

( ) Disagree

9. I can count on my neighbors for help:

( ) Agree

( ) I do not know, have no opinion

( ) Disagree

**Trust: school / neighborhood (score: 3 to 9)**

10. The teachers at my school are sympathetic and give us support:

( ) Agree

( ) I do not know, have no opinion

( ) Disagree

11. My neighbors would try to take advantage of me:

( ) Agree

( ) I do not know, have no opinion

( ) Disagree

12. My classmates would try to take advantage of me:

( ) Agree

( ) I do not know, have no opinion

( ) Disagree

**THANK YOU FOR HELPING US!!!**
